# Comparative Analysis of T4SS Molecular Architectures

**DOI:** 10.4014/jmb.2307.07006

**Published:** 2023-08-01

**Authors:** Mishghan Zehra, Jiwon Heo, Jeong Min Chung, Clarissa L Durie

**Affiliations:** 1 Department of Biochemistry, University of Missouri, Columbia, Missouri, USA; 2 Department of Biotechnology, The Catholic University of Korea, Bucheon-si 14662, Gyeonggi, Republic of Korea

**Keywords:** Type IV secretion system (T4SS), Dot/Icm, Cag, R388, cryo-EM

## Abstract

The recently published high-resolution R388 T4SS structure provides exciting new details about the complete complex of T4SS, including the components making up the stalk and arches, numerous symmetry mismatches between regions of the complex, and an intriguing interpretation of the closed stalk and radial symmetry of the inner membrane complex, which is related to pilus biogenesis assembly. However, there are a few unidentified densities in the electron microscopy map and portions of the identified component sequences for which the structure is not yet known. It is also unclear how well this minimized DNA-transporting T4SS predicts the structure of other T4SSs, such as expanded systems and those that transport proteins rather than DNA. In this review, we evaluate what can be inferred from the recent high-resolution structure of the R388 T4SS with respect to the Cag and Dot/Icm systems. These systems were selected because, given what is currently known about these systems, we expect them to present most structural differences compared to the R388 T4SS structure. Furthermore, we discuss bacterial physiology and diversity, the T4SS structures and their variations between different bacterial species. These insights may prove beneficial for researchers who elucidate the structure and functions of T4SS in different bacterial species.

## Introduction

Numerous bacterial species utilize complex secretion systems to transport macromolecules such as DNA or proteins across their cell membranes. These secretion systems are essential for bacterial virulence, survival, or both. Evolution has produced many different secretion systems, which have been grouped into families based on their similar features. One of the most versatile systems is the type IV secretion system (T4SS) family, which includes evolutionarily related but structurally and functionally diverse molecular complexes that can modulate bacterial interactions with their environments [1-3]. The T4SS family is unique in that its systems transport various substrates, including monomeric and oligomeric proteins, toxins, and nucleoprotein complexes [1, 2]. The substrates secreted by T4SSs mediate the conjugative transfer of mobile genetic elements among bacteria, which contributes to genome plasticity and confers antibiotic resistance, resulting in multidrug-resistant pathogens [1]. Furthermore, T4SSs directly contribute to bacterial pathogenicity by translocating macromolecular effectors into target eukaryotic cells, causing gastric cancer, peptic ulcers, pertussis, Legionnairés disease, and other various diseases in humans, animals, and plants [4]. Understanding the structure and function of T4SSs is crucial for the development of novel anti-virulence compounds and potential therapeutic applications that capitalize on the ability of T4SS to transfer macromolecules into targeted eukaryotic cells [5, 6]. In Gram-negative bacteria, the T4SS is made up of several components and embedded in two cell membranes, rendering high-resolution details difficult to obtain [1, 7]. However, decades of genetic, biochemical, and cell biology research, together with low- to moderate-resolution structural studies, have laid the foundation for understanding these systems.

T4SSs comprise a set of conserved core-complex subunits that span the inner and outer membranes of Gram-negative bacteria [8, 9]. In addition, T4SSs are found in Gram-positive bacteria; however, as their structures have not yet been thoroughly studied, they are outside the scope of this review. The prototypical T4SS is from *Agrobacterium tumefaciens* and is composed of 11 VirB and one VirD4 components. The 12 proteins that constitute this complex are organized into a 3-component (VirB7, VirB9, and VirB10) “core complex” that spans the inner and outer bacterial membranes, an inner membrane complex, three cytoplasmic ATPases, and other accessory or transiently associated components [1, 3, 9]. The architecture of many well-studied T4SSs, including those from the conjugation plasmids R388 and pKM101, and the plant pathogen *Xanthomonas citri*, are quite similar to those of *A. tumefaciens* Vir T4SS [10] based on sequence homologies, genetic determinants, and evolutionary relationships. These T4SSs are made up of 12 components with a diameter of approximately 200 Å and referred to as "minimized [6]." In contrast, expanded systems, which include the Dot/Icm T4SS from *Legionella pneumophila*, Cag T4SS from *Helicobacter pylori*, and F plasmid-encoded Tra T4SS, have a diameter of approximately 400 Å and comprise up to 30 components, with at least five components in the core complex [6].

The minimized and expanded classes of these mosaic nanomachines are organized as membrane-spanning apparatuses and form a transport conduit which, at least, includes an inner membrane complex (IMC) that connects to the outer membrane core complex (OMCC). Descriptions of these regions vary among species and researchers. At the base of the IMC, three ATPases provide energy to the system for translocation. In this large and diverse family, some systems also have an extracellular pilus, whereas other have additional subregions within the periplasmic space [11].

Genetic studies have identified components of the T4SS in some bacterial species, and biochemical studies have revealed the locations of these components with respect to the cytoplasm, and inner or outer membranes. Protein crystallography has been instrumental in determining the structure of individual components or small subregions of complexes in some minimized systems [12-20]. Negative stain electron microscopy (NS-EM) and moderate-resolution cryo-electron microscopy (cryo-EM) have been used to reveal the overall architecture of the systems [21-24]. However, until recently, the field lacked structural detail to understand the finer points of protein-protein interactions, regions of flexibility within the complex, and the basis for the versatility of secretion systems within the T4SS family. Recent advances in structural biology techniques, including single-particle cryo-EM and cryo-electron tomography (cryo-ET) of bacterial cells, have revealed unprecedented details regarding the molecular structures of these large, dynamic nanomachines [25-27]. Herein, we review the molecular architectures of expanded and minimized T4SSs, from the perspective of the first high-resolution reconstruction of a complete T4SS from the R388 plasmid.

## Key Insights from High Resolution R388 Structure

The recent publication of the complete T4SS structure from the R388 conjugative plasmid revealed important structural details at high resolution for the first time, greatly advancing our understanding of the molecular architecture of a T4SS [28]. The R388 plasmid is a well-characterized IncW plasmid isolated from *Escherichia coli*. IncW plasmids are less than 40 kb, are found in various species of *Enterobacteriaceae*, and carry different antibiotic resistance genes that are unidirectionally transferred to recipient bacteria, directly contributing to the rise of antibiotic-resistant bacterial strains [29]. R388 encodes for resistance to sulfonamide and trimethoprim, and its own secretion apparatus to transfer the plasmid, Trw T4SS [29, 30]. The Trw T4SS, which is highly homologous to VirB1-11/VirD4 T4SS in *A. tumefaciens*, is thought to operate in two modes: a pilus biogenesis mode, which requires only the VirB2-VirB11 components, and the DNA transfer mode, which requires these components and the ATPase coupling protein VirD4 [31].

**Fig. 1:**
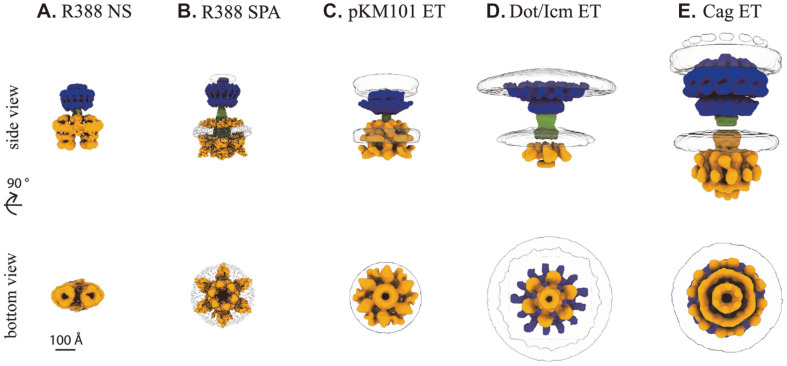
3D reconstructions of selected T4SS complexes. (**A**) The R388 T4SS determined by negative stain (NS) EM (EMD2567). (**B**) The R388 T4SS determined by single particle analysis (SPA) cryo-EM (EMD 13767, EMD13765). (**C**) The pKM101 T4SS determined by cryo-ET (ET) (EMD24100, EMD24098) (**D**) The Dot/Icm T4SS determined by cryo-ET (EMD7611, EMD7612). (**E**) The Cag T4SS determined by cryoET (EMD0634, EMD0635). In each reconstruction, the outer membrane core complex and periplasmic ring if applicable are shown in blue, the inner membrane complex including the arches or wings if applicable in orange, the stalk or cylinder connecting the outer membrane and inner membrane regions in green, and, if included, membrane density is shown as a transparent region with black outline.

Early NS-EM yielded an approximately 20 Å reconstruction of the VirB3-VirB10 complex isolated from the overexpression of an engineered plasmid containing R388 *virB1*-*virB10* with an affinity tag on *virB10* for purification in *E. coli* (Fig. 1A) [32]. The complex contains regions spanning from the inner membrane to the outer membrane. The OMCC is composed of an outer layer (O-layer) near the outer membrane and an inner layer (I-layer) in plane with the periplasmic space, with 14-fold radial symmetry and a diameter of approximately 185 Å. The IMC displayed a double-barrel organization with the longest dimension spanning approximately 225 Å. The OMCC and IMC are bridged by a thin density, referred to as the stalk. An additional density observed above the double-barrel structure of the IMC and the surrounding the stalk was identified as the arch. A second NS-EM reconstruction of this complex with the coupling protein VirD4 also exhibited a double-barrel-shaped IMC, with an additional density observed between the two barrels, indicating the position of VirD4 [33]. However, cryoET studies of other DNA-transporting T4SSs encoded by pKM101 (Fig. 1C) and the F conjugative plasmids exhibited radial symmetry in the IMC, rather than the double-barrel architecture [34, 35].

**Fig. 2:**
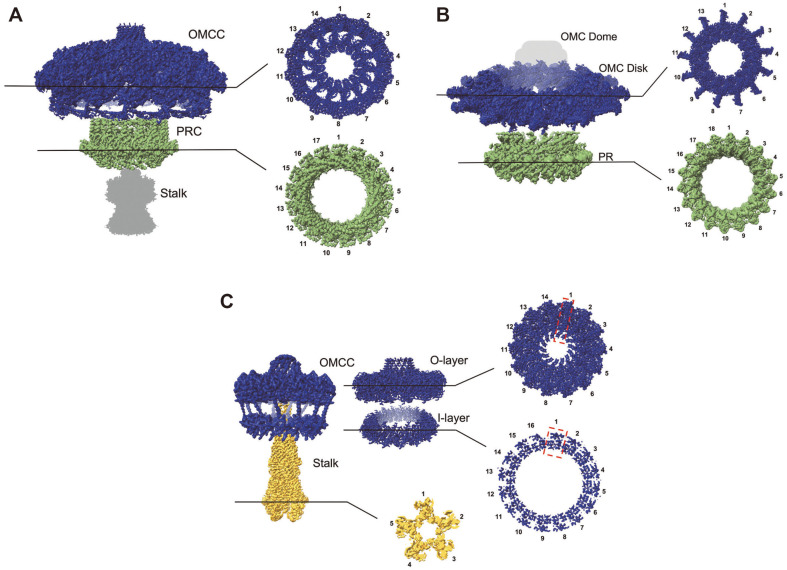
Symmetry mismatches in selected T4SS complexes. (**A**) The 3D reconstruction of the Cag T4SS reveals a distinct symmetry mismatch within its structure, composed of three elements: the Outer Membrane Core Complex (OMCC - EMD20020, blue) with 14-fold symmetry, the Periplasmic Ring Complex (PR - EMD20021, green) with 17-fold symmetry, and an uncharacterized stalk structure (grey). (**B**) The Dot/Icm T4SS exhibits its own unique symmetry inconsistencies. It comprises an OMC disk (EMD22068, blue) with a 13-fold symmetry and a PR (EMD22069, green) with an 18-fold symmetry. (**C**) Asymmetry is further illustrated in the 3D reconstruction of the R388 T4SS, which shows the OMCC (EMD13765, blue) composed of an O-layer (EMD12707) with a 14-fold symmetry, an I-layer (EMD12708) with a 16-fold symmetry, and a stalk (EMD13768, yellow) with a 5-fold symmetry.

A recent single-particle cryo-EM study produced the first high resolution (3-6 Å) structure of the intact VirB3-10 T4SS from R388 (Fig. 1B) [28]. The detailed OMCC structure was largely consistent with the well-characterized OMCCs from minimized T4SS, including the identity and positioning of VirB7, VirB9, and VirB10 [12, 36]. However, there was one exception: while the R388 OMCC O-layer exhibited the previously observed 14-fold radial symmetry, the I-layer exhibited 16-fold symmetry (Fig. 2C) [28]. This symmetry mismatch within the OMCC has not been identified in previous structural studies of the R388 T4SS [32]. VirB9 was identified as the component spanning the symmetry mismatch with the C-terminal domain in the O-layer and the N-terminal domain in the I-layer, with two additional VirB9 N-terminal domains in the I-layer compared to the O-layer [28].

The narrow stalk is inserted into the periplasmic side of the OMCC, and here the authors revealed the first high resolution structure of this region (Fig. 2C) [28]. This region appears to be flexible, with the relative positioning of the OMCC and IMC varying substantially at either end. The stalk exhibits 5-fold radial symmetry, which has a different degree of symmetry than that observed in either the OMCC or IMC. The stalk is a cone-shaped pentamer with VirB6 forming the base inserted into the inner membrane, and VirB5 forming a point extending up into the OMCC. Although there were no previous structures of the stalk, VirB5 homologues from other systems were isolated and their structures were determined by X-ray crystallography [15, 37]. Here, the N-terminus extends up in the complex making the narrowest part of the cone, which is a markedly different conformation compared to the structures of homologues in isolation in which the N-terminus is compact and in-plane with the globular fold of the protein [15, 28, 37].

The region described as the arches has also been poorly understood, with no information previously available on the protein components in this region or the symmetry present [23, 33]. The recent study revealed, for the first time, that a hexamer of trimers of the VirB8 periplasmic domain forms a ring around the stalk [28]. The subunits in each trimer present different protein-protein interactions. The interface between the first and second protomers resembles that of *H. pylori* CagV, but the interface between the second and third protomers is similar to that of *Brucella* VirB8, where each paralog structure is a crystal structure of truncated constructs that forms dimers [20, 38].

In the recent cryo-EM structure [28], IMC was in the form of a large hexameric ring, similar to that observed in cryoET [34, 35], rather than the double-barrel structure observed in previous NS-EM studies (Figs. 1A and 1B)[32, 33]. Notably, the protein components and their stoichiometries were the same, whereas their arrangements were drastically different. VirB4 was the main component, and 12 copies were arranged in a hexamer of dimers with a central ring. An approximately 4 Å structure of VirB4 was separately determined and then fitted into the reconstruction of the complex because of the lower resolution in this region, which may be related to the variable occupancy of the VirB4 ATP-binding sites. Free VirB4 oligomerizes into a ring described as a “trimer of dimers.” This trimer of dimers is similar in size and shape to one barrel in the double-barrel structure previously observed [32, 33]. Two-dimensional classes were observed in this dataset, which corresponded to the double-barreled IMC structure; however they comprised a minority (0.3%) of particles [28]. This suggests that there could be different forms or assemblies of the IMC, consistent with the idea that there are different active conformations, such as a pilus assembly and a DNA transfer complex. For each VirB4 dimer, one VirB3 makes significant contacts with the central VirB4, and three copies of the N-terminus of VirB8 are associated to varying degrees with the outer VirB4, resulting in the IMC stoichiometry of 1:2:3 (VirB3:VirB4:VirB8).

Many secretion systems have a continuous central channel that extends from the inner to the outer membrane, a pathway through which cargo is exported from the cell [39, 40]. However, there was no continuous central channel in the observed structure [28]. The stalk blocks the path that may have existed from the central opening of the IMC to the outer membrane opening of the OMCC. Researchers have suggested that this structure represents the R388 T4SS in its pilus biogenesis state [28].

VirB4 is one of three ATPases known to be essential for T4SS functioning. VirD4, the coupling complex ATPase known to transport single-stranded DNA through the T4SS, is not visualized in this structure, although previous low-resolution EM revealed that it can bind between the two barrels of the IMC when in that conformation (Fig. 1A) [33]. If we hypothesize that this hexamer conformation of IMC dimers represents a pilus biogenesis assembly, it is reasonable that VirD4 is not engaged. Next, we considered what is known about the third ATPase, VirB11. The authors sought to address this question using two approaches. First, interactions between VirB4 and VirB11 were predicted using AlphaFold and validated by analyzing the co-evolution of residues at the interface using TrRosetta [28]. Second, the pKM101 plasmid homologues TraB and TraG, were co-purified with and without single point mutations at selected predicted interface residues, and weaker interactions were observed when a mutation was present compared to the wild-type proteins [28]. This suggests that VirB11 likely binds to the central VirB4 hexamer.

The high-resolution R388 T4SS structure provides exciting new details about the complete complex, including the components making up the stalk and arches, numerous symmetry mismatches between regions of the complex, and an intriguing interpretation of the closed stalk and radial symmetry of the IMC, which is related to pilus biogenesis. However, several questions remain. There are a few unidentified densities in the EM map, and portions of the identified components sequences for which the structure is not yet known. Most importantly, this structure invites efforts to characterize the active transport conformation of R388 T4SS. It is also unclear how well this minimized, DNA-transporting T4SS predicts the structure of other T4SSs, such as expanded systems and those that transport proteins rather than DNA.

## Protein-Translocating, Expanded T4SS Structures

Here, we assess the known information regarding expanded protein-translocating T4SSs from the human pathogens *H. pylori* and *L. pneumophila*. Despite transporting only the oncoprotein CagA, *H. pylori* Cag T4SS belongs to the same subfamily as the R388 T4SS; this is type IVA secretion systems (T4ASS), which is defined by similarity to the system in *A. tumefaciens* [41]. *L. pneumophila* Dot/Icm T4SS is thought to translocate more than 300 protein substrates and is more distantly related to the Cag and R388 T4SSs [42, 43]. The Dot/Icm system is a type IVB secretion system (T4BSS), a subfamily that is genetically similar to the IncI plasmid [44]. The Cag and Dot/Icm systems are both considered expanded systems because they comprise approximately 17 and 30 protein components, respectively, and five or more core components, compared with 12 protein components and three core components in minimized systems. Excellent reviews have recently compared these and other T4SSs including those from *X. citri*, the pKM101 plasmid, and the F conjugative plasmid [6, 26]. Here, we evaluate the recent high-resolution structure of R388 T4SS in comparison with Cag and Dot/Icm systems. Recent advancements have shaped a profound understanding of how the Cag and Dot/Icms T4SSs organize into a multi-protein channel. Thus, these two systems were selected for comparative analysis because we expected to observe significant structural differences regarding the R388 T4SS. Moreover, the conserved features among these systems could be envisioned as potential targets for the development of novel anti-virulence compounds to treat polymicrobial infections and avert the crisis of antibiotic resistance.

### OMCC

As described above, the OMCC of R388 T4SS (T4SS_R388_ and OMCC_R388_) is composed of VirB7, VirB9, and VirB10, forming a barrel-shaped structure with a diameter of 185 Å. The OMCC_R388_ O-layer has 14-fold symmetry and 1:1:1 stoichiometry within the asymmetric unit, whereas the I-layer has 16-fold symmetry and 1:1 stoichiometry of VirB9 and VirB10 [28]. The more closely related Cag T4SS (T4SS_Cag_) also has an OMCC O-layer with 14-fold symmetry but with five components in a stoichiometry of 1:1:2:2:5 (CagY:CagX:CagT:CagM:Cag3) per asymmetric unit [45, 46]. Three of these proteins, CagT, CagX, and CagY, are homologous to the T4SS_R388_ components VirB7, VirB9, and VirB10, respectively, whereas CagM and Cag3 are specific to T4SS_Cag_ [45, 46]. OMCC_Cag_ also comprises of an O- and I-layer, with no symmetry mismatch between these layers. The overall size and shape were quite different from those of T4SS_R388_. OMCC_Cag_ has a diameter of approximately 400 Å, and a shape more like a mushroom cap than a barrel (Figs. 1E and 2A) [45].

The Dot/Icm OMCC (OMCC_Dot/Icm_) is also comprised of the approximate homologues of VirB7, VirB9, and VirB10 [4, 47]. In Dot/Icm T4SS (T4SS_Dot/Icm_), DotD, DotH, and DotG, exhibit similar folds to VirB7, VirB9, and VirB10, despite low sequence similarity [4, 47]. Six additional species-specific components were also identified. DotC and DotF complete the five predicted core components based on previous biochemical studies [4, 47]. DotK has also been associated with the OMCC [4, 47]. Additionally, three proteins not previously known to be part of T4SS_Dot/Icm_ were identified by high-resolution structural determination and named Dis1, Dis2, and Dis3 (Dot/Icm secretion) [47]. OMCC_Dot/Icm_ has a diameter similar to that of OMCC_Cag_, at approximately 400 Å [4, 22, 47]. Unlike OMCC_Cag_ and OMCC_R388_, the DotG (VirB10) C-terminal domain forming the outer membrane of the pore is discontinuous with the rest of the OMCC; therefore, this pore- forming region is referred to as the dome, whereas the flat portion is referred to as the disk [4,47]. The disk and dome displayed different degrees of symmetry (13-fold and 16-fold, respectively), a feature not observed in OMCC_Cag_ and OMCC_R388_ [47]. OMCC_Dot/Icm_ does not have an O- and I-layer, but rather a flat disk region. The dome is made up of 16 copies of the C-terminus of DotG[47]. The stoichiometry of the disk within an asymmetric unit was 2:1:1:1:1:1:1 (DotD:DotC:DotF:DotK:Dis1:Dis2:Dis3)[47].

### PR

The periplasmic ring (PR) is a feature observed in the expanded T4SSs. While T4SS_R388_ has no PR[28, 32, 33], both T4SS_Cag_ and T4SS_Dot/Icm_ exhibit PRs (PR_Cag_ and PR_Dot/Icm_ respectively) [4, 7, 45, 47-49]. PR_Cag_ and PR_Dot/Icm_ display different degrees of symmetry, 17-fold and 18-fold, respectively (Figs. 2A and 2B) [4, 45, 47]. Both are comprised of their respective homologues of the VirB9 N-terminal domain and a species-specific domain of the VirB10 homologue in a 1:1 stoichiometry. PR_Dot/Icm_ also includes a second copy of the species-specific component DotF and an additional protein density that could not be identified [47]. The I-layer of OMCC_R388_ is comprised of VirB9 and VirB10 N-terminal domains and displays a symmetry mismatch with the O-layer; this could be considered similar to PR in some respects [28]. Therefore, structures from this diverse family reveal architectures that exist along a spectrum of organizational motifs rather than discrete structural classes.

### Stalk

The stalk regions of the Cag and Dot/Icm systems (Stalk_Cag_ and Stalk_Dot/Icm_, respectively) have been observed by cryoET of bacterial cells [7, 48, 49]. However, this region of the complex appears to dissociate upon extraction for single-particle cryo-EM analysis, with only weak density observed in T4SS_Cag_, and this region was not observed in T4SS_Dot/Icm_ single-particle reconstructions [4, 45, 47]. Therefore, the symmetry of this region in T4SS_Cag_ and T4SS_Dot/Icm_ remains unknown. The VirB5 and VirB6 proteins that comprise the stalk in T4SS_R388_ have homologues in H.pylori, CagL, and CagW, which are implicated in pilus formation, as are VirB2 and VirB5 [50, 51]. However, while the cone-shaped density inserted into PR_Cag_ observed in single-particle reconstructions is referred to as the stalk, *in situ* cryo-ET revealed a hollow cylinder of consistent width spanning the distance from the IMC to the OMCC [48]. There may be various functional conformations of the T4SS_Cag_ complex, analogous to the pilus biogenesis and DNA-transfer complexes proposed for T4SS_R388_.

However, T4SS_Dot/Icm_ does not have a pilus. Furthermore, there are no predicted homologues of the VirB5 and VirB6 proteins that make up the stalk in the T4SS_R388_ in *L. pneumophila*. *In situ* cryo-ET suggests that the stalk is composed of a portion of DotG [52]. The C-terminus of DotG is homologous to that of VirB10; however, DotG is a much larger protein and exhibits low sequence similarity to components of other T4SSs. This region may have unique domains, as 16 copies of the DotG C-terminal domain were observed in the OMCC_Dot/Icm_ dome and 18 copies of DotG (residues 791-824) were observed in the PR_Dot/Icm_ [47]. If DotG comprises the stalk, the number of copies involved cannot be predicted.

### Arches

Additional density around the stalk was observed in the *in-situ* studies of both T4SS_Cag_ and T4SS_Dot/Icm_. These densities have been described as wings or a collar structures [48, 52]. Similar to the arches of T4SS_R388_, the additional densities were above the IMC and appeared to surround the stalk; however, they did not remain associated with the core complex during extraction from the membrane for single-particle analyses. The architecture of these regions, as observed by cryo-ET revealed a different orientation than that of T4SS_R388_. The T4SS_R388_ arches extend horizontally above the inner membrane and then curve toward the IMC, so that the widest part of this region is within the inner membrane [28]. In contrast, the wings or collars of the expanded T4SSs are narrow above the inner membrane and extend outward as they reach further into the periplasmic space toward the outer membrane [48, 49, 52]. The T4SS_Dot/Icm_ wings are thought to comprise the species-specific protein DotF [52], whereas the composition of the collar of the T4SS_Cag_ remains undefined.

### IMC and Coupling Complex

Similar to the stalk and arches, IMC dissociated from the T4SS_Cag_ and T4SS_Dot/Icm_ during extraction. However, its architecture has been observed in *in-situ* studies [48, 53, 54]. The IMC in both T4SS_cag_ (IMC_cag_) and T4SS_Dot/Icm_ (IMC_Dot/Icm_) was observed as a series of concentric rings with six-fold symmetry comprised of at least two of the three conserved ATPases, which is comparable to the recent T4SS_R388_ structure (Figs. 1B, 1D, and 1E).

The IMC_Dot/Icm_ was similar to that of T4SS_R388_ (IMC_R388_) observed in the recent structure determined from single particle analysis cryo-EM [53, 54]. In the IMC_Dot/Icm_, the VirB4 homologue DotO forms a hexamer of dimers with its central ring aligned with the center of T4SS_Dot/Icm_ [54]. A hexameric ring of the VirB11 homologue, DotB, binds to the central ring of DotO on the cytoplasmic side, as predicted in the recently determined T4SS_R388_ structure [28, 54]. The VirD4 homologue, DotL, has not been observed to interact with IMC_Dot/Icm_, but is rather part of a separate subassembly, the coupling complex. The coupling complex was isolated and determined to be composed of DotL, DotM, and DotN, the recently discovered components DotY and DotZ, and under some conditions, IcmS, IcmW, and LvgA [55]. This complex forms a hexameric ring with the transmembrane domains of DotL and DotM, which are thought to form pores in the inner membrane [55]. The interaction between this coupling complex and IMC_Dot/Icm_ remains unknown.

IMC_Cag_ observed by cryoET, like IMC_R388_ and IMC_Dot/Icm_, exhibits a hexamer of dimers of the VirB4 homologue CagE at the inner membrane, with its central channel aligned with the center of the core complex [48]. The authors described this as an “inverted V” when viewing a cutaway side view of the 3D surface rendering from subtomogram averages [48]. The VirB11 homologue Caga forms a hexameric ring that binds to the central ring of CagE, similar to the homologues observed in IMC_Dot/Icm_ and predicted in IMC_R388_ [28, 48, 54]. Remarkably, however, the VirD4 homologue Cagb interacts with CagE and Caga in a conformation not observed in other T4SSs [48]. Here, Cagb formed three concentric rings. In the inner ring, Cagb was associated with Caga. In the middle ring, Cagb associates with the outer ring of CagE. In the outer ring, Cagb associates with to the inner membrane [48]. In contrast, studies of T4SS_R388_ have shown that VirD4 forms hexameric rings between the double barrels of the IMC in a putative DNA transport conformation [33], while studies of T4SS_Dot/Icm_ have yet to reveal DotL or the larger coupling complex associated with the core complex [49, 52, 54, 55]. Additionally, biochemical and genetic studies studies have predicted that the VirB6 homologue CagW and the VirB8 homologue CagV both have polytopic transmembrane regions and may be part of the IMC_Cag_; however, they have not yet been visualized in structural studies [48, 50, 56].

## Structural Variations and Cargo Transport in T4SSs

The structural differences among the T4SS_R388_, T4SS_Dot/Icm_, and T4SS_Cag_ systems have evolved to serve specific functional roles in bacterial processes [6]. Many of the structural features of the minimized T4SSs are conserved within the extended T4SSs [8, 9], particularly the components of the core complex, suggesting that it is central to the assembly process of the entire T4SS complex [26]. Both minimized and expanded T4SSs exhibit a symmetry mismatch between each subcomplex, which provides flexibility between each layer [4, 28, 45-47, 57]. The biological function and mechanisms of this structural mismatch have not yet been elucidated; however, its presence in other bacterial secretion systems, such as T2SS, T3SS, and T6SS, suggests its importance [39, 58, 59].

Across different bacterial species, T4SSs exhibit structural differences due to species-specific components and stoichiometric variations in subassembly components, leading to architecture diversification and contributing to functional diversity [26, 45-47]. For instance, the core complex of the extended T4SSs, such as T4SS_Cag_ of *H. pylori* and T4SS_Dot/Icm_ of *L. pneumophila*, has a much larger diameter than that of the minimized system [26]. In addition, the T4SS complex of certain species is characterized by the presence of a pilus, whereas others lack this feature [9, 34, 41, 52, 60-63]. The pilus is a filamentous structure that extends from the bacterial cell, enabling direct cell-to-cell contact. Structural differences among species can be linked to the various functional characteristics of T4SS. Pilus-mediated DNA transfer of T4SS_R388_ is crucial for its role in bacterial conjugation. The VirB11 ATPase is involved in the dynamic regulation of pilus assembly and disassembly, as well as in the energization of the translocation process [28, 64, 65]. The VirD4 coupling protein recruits and prepares the DNA substrate for translocation, interacts with relaxase-bound DNA, and guides it to the T4SS [31]. The structural organization of the T4SS_R388_ components ensures a stable and efficient DNA transfer, contributing to the spread of antibiotic resistance and virulence factors among bacteria.

In contrast, the T4SS_Dot/Icm_ system evolved to deliver multiple effector proteins into host cells, which is essential for the intracellular survival and replication of pathogens, like *L. pneumophila*. The complex substrate recognition and recruitment strategy employed by T4SS_Dot/Icm_ allows it to handle a diverse array of effector proteins, recognizing and transporting a large number of substrates without being limited by a specific coupling protein. This unique mechanism, which involves Dot/Icm proteins (DotB, IcmQ, IcmR, IcmS and IcmW), enables the system to recognize and transport a large number of substrates [66-68]. The intricate composition of the T4SS_Dot/Icm_ OMCC contributes to its versatility, accommodating different substrates [68].

The T4SS_Cag_ system, found in *H. pylori*, has evolved unique features that ensure effective delivery of the CagA effector protein to host cells. CagY, which shares homology with VirB10, forms an extended structure that projects beyond the bacterial cell surface [41, 45, 46]. This structure is thought to facilitate direct contact with the host cells, enhancing the efficiency of CagA translocation. Additionally, CagY has been implicated in the regulation of T4SS_Cag_ activity through phase variation, which allows *H. pylori* to modulate its interaction with the host immune system [60, 69-71]. The T4SS_Cag_ machinery comprises several components that share homology with those of other T4SSs; however, it also includes unique proteins, such as Cag3 and CagM, which are essential for its function and CagA translocation [41]. In addition to the CagA effector protein, the T4SS_Cag_ system is responsible for the translocation of peptidoglycan fragments, which are recognized by the host cell's NOD1 receptor [72, 73]. This interaction triggers an inflammatory response, contributing to the development of gastric diseases such as gastritis, peptic ulcers, and gastric cancer [74]. The structural adaptations of the T4SS_Cag_ system not only facilitate efficient effector protein translocation but also enable the manipulation of host cell processes through the delivery of additional virulence factors. The T4SS_Cag_ pilus is suggested to be more adaptable than the T4SS_R388_ pilus, potentially allowing it to adjust to different host cell environments [55, 61, 62, 68, 75]. This flexibility may be crucial for the ability of *H. pylori*'s to colonize diverse host cell types in the human stomach, where it encounters a wide range of physiological conditions.

## Conclusion

This review compares the architectural features of selected T4SS assemblages that provide exciting new insights into the structural organization of T4SSs [4, 13, 15, 35-37, 39, 43]. The structural differences among T4SS_R388_, T4SS_Dot/Icm_, and T4SS_Cag_ highlight the various adaptations that have evolved to suit their specific functional roles. T4SS_R388_ is optimized for bacterial conjugation, whereas T4SS_Dot/Icm_ complex substrate recognition strategy allows it to transport multiple effector proteins into host cells, promoting intracellular survival and replication [4, 15, 37]. The unique features of T4SS_Cag_, such as the extended CagY structure and the potentially more adaptable pilus, enhance the delivery of the CagA effector protein, thereby modulating host cell processes and facilitating *H. pylori* colonization [31, 35-36, 39]. Understanding these structural adaptations offers valuable insights into the diverse and specialized roles of T4SSs in bacterial biology and pathogenesis. Additionally, it highlights the potential of targeting these systems for the development of novel antimicrobial strategies, as the disruption of T4SS function could impair bacterial conjugation, virulence factor delivery, and colonization of host cells. However, despite these advances, these model systems still require further elaboration to solve the long-standing concerns regarding the mechanism of effector processing and translocation, signals involved in the recruitment and recognition of effectors with consequent activation of the channel, and structural adaptations leading to functional versatility. Therefore, further in-depth structural studies for each T4SS are required to address these concerns and integrate the knowledge of the structure into a translational approach for designing structure-associated inhibitors or using these nanomachines as therapeutic vehicles.
